# A Novel Hierarchically Porous Polypyrrole Sphere Modified Separator for Lithium-Sulfur Batteries

**DOI:** 10.3390/polym11081344

**Published:** 2019-08-13

**Authors:** Baoe Li, Zhenghao Sun, Yan Zhao, Zhumabay Bakenov

**Affiliations:** 1 School of Materials Science and Engineering, Hebei University of Technology, Tianjin 300130, China; 2 Institute of Batteries LLC, National Laboratory Astana, Nazarbayev University, 53 Kabanbay Batyr Avenue, Nur-Sultan 010000, Kazakhstan

**Keywords:** lithium-sulfur batteries, separator, shuttle effect, hierarchically porous polypyrrole sphere, electrochemical performance

## Abstract

The commercialization of Lithium-sulfur batteries was limited by the polysulfide shuttle effect, and modifying the routine separator was an effective method to solve this problem. In this work, a novel hierarchically porous polypyrrole sphere (PPS) was successfully prepared by using silica as hard-templates. As-prepared PPS was slurry-coated on the separator, which could reduce the polarization phenomenon of the sulfur cathode, and efficiently immobilize polysulfides. As expected, high sulfur utilization was achieved by suppressing the shuttle effect. When tested in the lithium-sulfur battery, it exhibited a high capacity of 855 mAh·g^−1^ after 100 cycles at 0.2 C, and delivered a reversible capacity of 507 mAh·g^−1^ at 3 C, showing excellent electrochemical performance.

## 1. Introduction

Lithium-sulfur (Li-S) batteries, which possess superior theoretical specific capacity (1672 mAh·g^−1^), is regarded as one of the most promising rechargeable batteries [1,2,3,4]. However, Li-S batteries still cannot satisfy the needs of practical applications, for example, the low conductivity of sulfur and polysulfide intermediates lead to low utilization efficiency, especially cycled at a high current rate. Furthermore, the unavoidable intermediate lithium polysulfides; Li_2_S*_x_* (4 ≤ *x* ≤ 8) could lead to the gradual loss of the sulfur, as well as a serious fade in the capacity [5,6,7,8,9,10,11,12]. Therefore, nanostructured host materials, including carbon materials, polymers and metal oxides, have been designed to confine sulfur or its intermediates and improve the conductivity [13,14,15,16].

Presently, the composite modified separator has been found as one of the effective methods to alleviate the solution of polysulfides for improving the electrochemical performance [17,18,19,20,21]. Various conductive materials have been used as the coating layer and introduced between the cathode and separator, not only to block the shuttle effect of the polysulfide, but also to suppress the volume expansion of the sulfur [22,23,24,25]. For example, conductive metal oxides, carbon materials, and conductive polymers have been used as intermediate layers for suppressing the shuttle effect. Among them, polypyrrole (PPy) was considered a superior polysulfide immobilizer because of its good electrical conductivity and stable physicochemical properties [26,27,28,29,30].

In Li et al′s study, a polypyrrole coated separator was used to suppress the shuttle effect, and when the cell was equipped with such a modified separator, it exhibied a stable cycling performance [31]. Ma et al designed a self-assembled polypyrrole nanotube film (PNTF), as a functional interlayer for Li-S cells. The resulting cell showed an encouraging electrochemical performance, due to that PPy having a strong interaction with polysulfides [32]. In Yin et al′s work, a novel modified separator was prepared by coating PPy/ZnO composite slurry on a routine separator. Due to the polar ZnO and porous polypyrrole, PPy/ZnO composites could act as polysulfide immobilizers to intercept the migration of soluble polysulfides [33].

Having this mind, in this work, we developed a novel hierarchically porous polypyrrole sphere (PPS) modified separator for Li-S cells and investigated their electrochemical performance. PPS was designed and prepared via a hard-template method because we simultaneously integrated the physical encapsulation of the rich mesopore in PPS and the on-site chemical adsorption PPS to polysulfides into a coating layer system. This novel optimized configuration presented here is potentially suitable for application in future Li-S cells.

## 2. Materials and Methods 

The silica (SiO_2_) template nanosphere (with a diameter of ~30 nm) was prepared by the method reported [34,35]. 0.5 g of template was added to the resulting solution (20 mL) with 0.2 mM pyrrole and 0.2 mM sodium acetate, and then the mixture was stirred for 6 h. The mixture and FeCl_3_ solution (0.2 mM, 20 mL) were placed for stirring for 4 h. After reacting, the precipitates were washed with deionized water and dried at 60 °C. Subsequently, the PPS/SiO_2_ was etched using an HF (10 wt %, 72 h) at 60 °C. Then, the PPS was obtained after filtrating and washing with distilled water. For preparation of PPS-modified separator, 80 wt % PPS, 10 wt % super-P, and 10 wt % polyvinylidene fluoride (PVDF) were mixed in N-methylpyrrolidinone (NMP) to form a slurry which was then cast on the routine separator. The sample was dried out and cut into circular disks with a mass loading of 0.35 mg·cm^−2^. 

The samples were analyzed by X-ray diffraction (XRD, D8 Discover Bruker, Billerica, MA, USA) and Fourier-trans-form infrared spectroscopy (FT-IR, TENSOR, Billerica, MA, USA). The specific surface area and pore size distributions were analyzed by N_2_ adsorption-desorption isotherms measurement (V-Sorb 2800P, Gold APP, Beijing, China) and calculated based on multipoint Brunauer–Emmett–Teller (BET) and Barrett–Joyner–Halenda (BJH) methods. The morphology and microstructure were measured using scanning electron microscopy (SEM, JSM-7600F, JEOL Ltd., Akishima, Japan), and transmission electron microscopy (TEM, JEOL-2100, JEOL Ltd., Akishima, Japan). The elemental composition was analyzed by X-ray photoelectron spectroscopy (XPS, K-Alpha 1063, Thermo Fisher Scientific, Waltham, MA, USA).

The sulfur cathode composited of sulfur (70 wt %), super-P (20 wt %), and PVDF binder (10 wt %) was dissolved in NMP. The resulting slurry was uniformly coated on aluminum foil and dried at 60 °C in vacuum for 12 h. The sulfur cathode was cut into circular disks and the sulfur loading was 2.1 mg·cm^−2^. The PPS-modified separator was assembled in the electrolyte made of 1 M lithium bistrifluoromethanesulfonamide (LiTFSI) mixed with 1,3-dioxolane (DOL) and dimethoxy ethane (DME) with 1:1 volume ratio. The CR2025 coin cells were assembled in argon-filled glove box and tested using a battery test instruments (NEWARE BTS-4000, Shenzhen, China). The electrochemical measurement system (Princeton Applied Research, Versa STAT4, Oak Ridge, TN, USA) was used for Cyclic voltammetry (CV) and electrochemical impedance spectrometry (EIS) measurement.

## 3. Results

**Figure 1:**
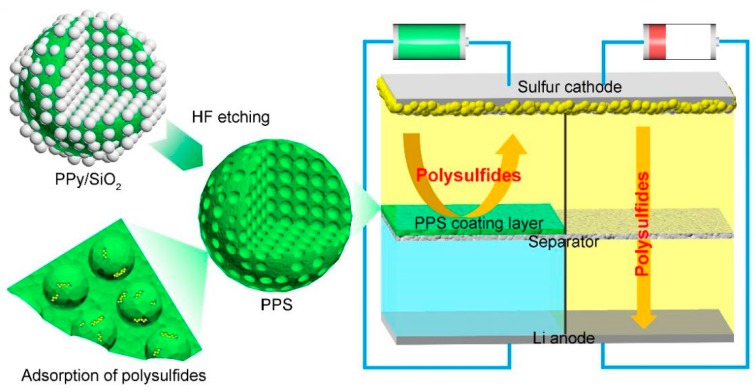
Schematic of PPS structural characteristics and Li-S cell with PPS-modified separator.

The PPS structural characteristics and the schematic diagram of Li-S batteries equipped with PPS-modified separator were shown in Figure 1. The porous PPS coating layer effectively blocks the shuttle effect of polysulfide, improving the electrochemical performance of Li-S batteries. 

**Figure 2:**
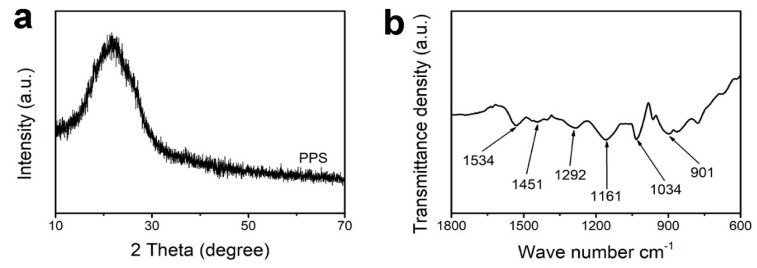
(**a**) XRD pattern and (**b**) FT-IR spectrum of PPS.

Crystal microstructural properties of the PPS were investigated by XRD analysis in Figure 2a. A broad characteristic peak located at about 24° was founded, corresponding to the successful formation of the polypyrrole material [36]. The FT-IR spectrum of PPS showed several obvious characteristic bands in Figure 2b. The bands of PPS located at 1534 and 1451 cm^−1^ were ascribed to the pyrrole ring basic vibrations. The bands of 1292 and 1034 cm^−1^ were assigned to the C–H in-plane vibration and the adsorption band at 1161 cm^−1^ was attributed to C–N stretching vibration [37]. 

**Figure 3:**
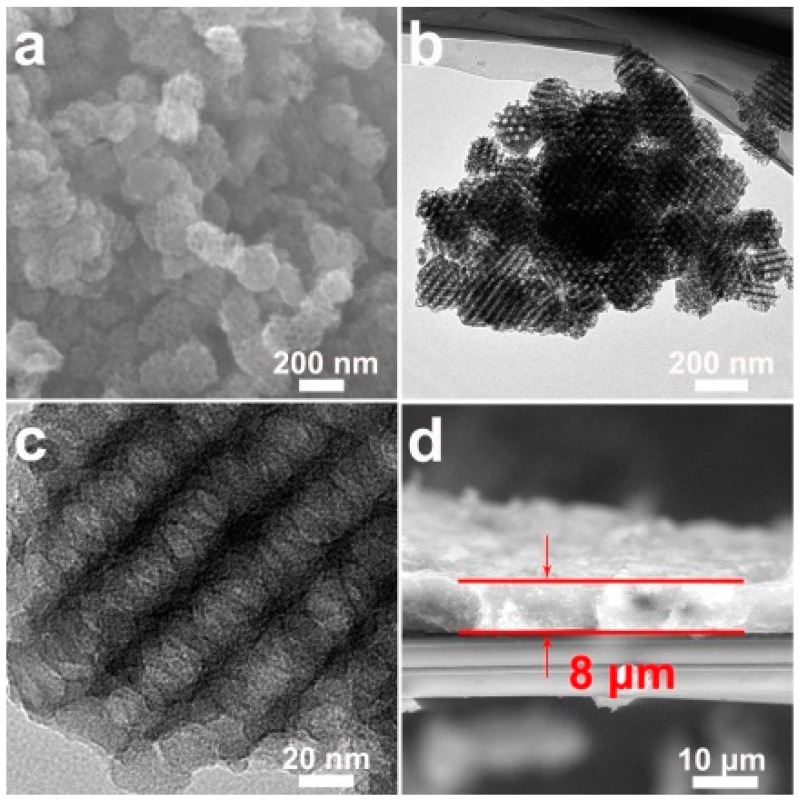
(**a**) SEM image of PPS; (**b**,**c**) TEM images at different magnifications of the PPS; (**d**) cross-section SEM image of PPS coating layer.

The morphology and microstructure of PPS were measured by SEM and TEM (Figure 3). As shown in Figure 3a, PPS exhibited a spherical matrix structure, and a three-dimensional ordered spherical mesopore was present in the PPS matrix. At the same time, TEM images were employed to examine the internal structural features of the PPS. The TEM image showed the mean mesopore size was about 30 nm in Figure 3b. In addition, the image of Figure 3c delivered the interconnected mesoporous structure for facilitating the transmission [38]. The PPS was designed hierarchically as the coating layer could effectively trap polysulfides to restrain the shuttle effect, and enhance the cycling performance of Li-S cells. The PPS was applied onto one side of the routine separator, yielding an 8 µm thick film, as shown in Figure 3d.

**Figure 4:**
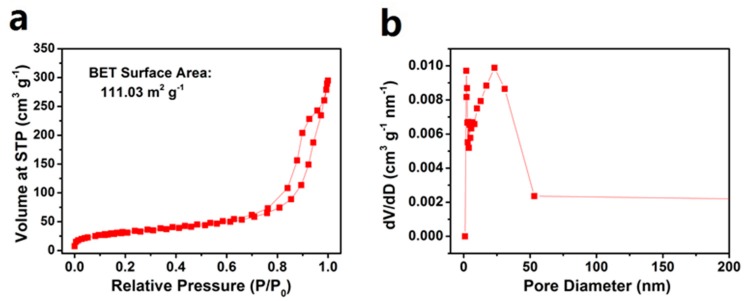
(**a**) N_2_ adsorption/desorption isotherms and (**b**) Pore size distribution of the PPS.

Figure 4a exhibits the N_2_ adsorption-desorption isotherms for the PPS with a large BET surface area of 111.03 m^2^·g^−1^. The average pore size is about 2.71 nm, indicating that the small mesopores were embedded in the PPS (Figure 4b). The results of the investigation of the pore size distribution for PPS were in agreement with the TEM images.

**Figure 5:**
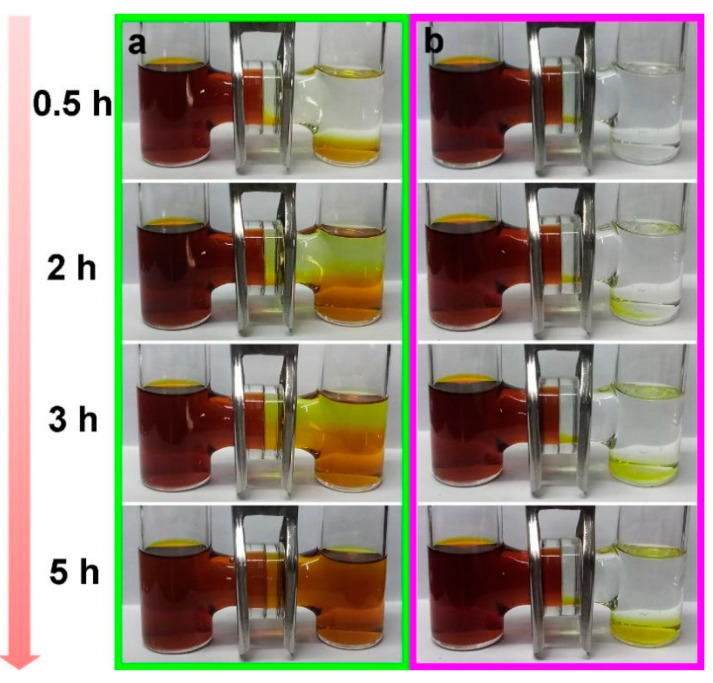
Polysulfide permeation measurements for (**a**) routine separator and (**b**) PPS modified separator.

In order to visually prove that the PPS modified separator can effectively suppress shutting effect, the polysulfide penetration test was carried out in H-type bottles. The left half of the bottle contained 0.1 M Li_2_S_6_-THF solution, and the blank THF solution was filled in the right half of the bottle, and the bottles were separated by the routine separator or the PPS modified separator. Obviously, the color of the right bottle equipped with the routine separator gradually turned reddish-brown (Figure 5a), indicating a severe polysulfide penetration phenomenon. In contrast, as shown in Figure 5b, the bottle equipped with the PPS modified separator exhibited almost no color change even after five hours, exhibited an effective block to polysulfide [39].

**Figure 6:**
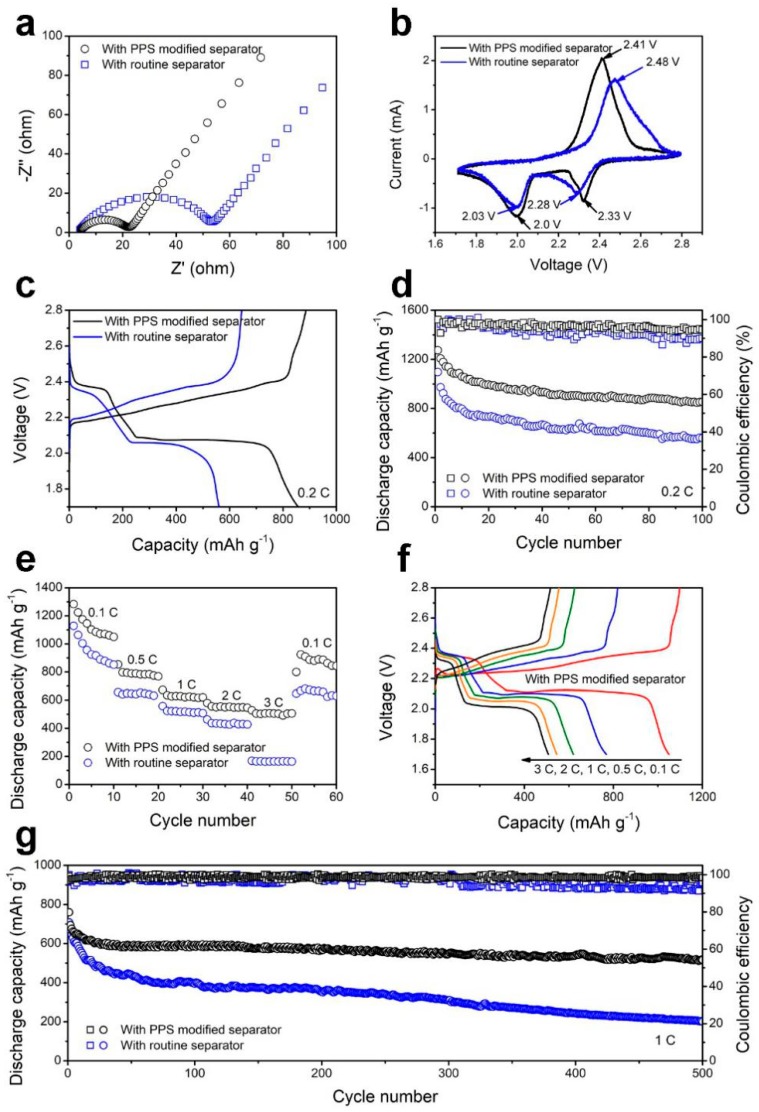
(**a**) EIS spectra; (**b**) CV profiles; and (**c**) charge-discharge curves of Li-S cells with the PPS-modified separator and the routine separator; (**d**) Cycling performance of Li-S cells with the PPS-modified separator and the routine separator at 0.2 C; (**e**) Rate performance of Li-S cells with the PPS-modified separator and the routine separator; (**f**) Charge-discharge curves of Li-S cells with the PPS-modified separator at various rates; (**g**) Long-term cycling performance of Li-S cells with the PPS-modified separator and the routine separator at 1 C.

The EIS measurements were carried out as illustrated in Figure 6a. The cell with the PPS-modified separator shows a smaller charge transfer resistance than with the routine separator, which can be attributed to the superior conductivity of the PPS coating layer [40]. Figure 6b shows the CV profiles of the cells with different separators. Obviously, typical oxidation and reduction peaks of cells were visible. The cell with the PPS-modified separator showed two cathodic peaks located at 2.33 and 2.0 V, corresponding to a reduction of sulfur to polysulfide and finally to Li_2_S_2_ (or Li_2_S), respectively [41]. The anodic peak with the PPS-modified separator were located at around 2.41 V, corresponding to the oxidation of Li_2_S_2_ (Li_2_S) to sulfur [42]. Meanwhile the cell with the routine separator exhibited two cathodic peaks (2.28 and 2.03 V) and one anodic peak (2.48 V) as well, which revealed the peaks shift. The charge-discharge curves corresponded to the CV results. The charge-discharge curves of the cell with the PPS-modified separator showed a higher reduction plateau and lower oxidation plateau than those with the routine separator, which was ascribed to the lower polarization (Figure 6c). After 100 cycles, the cells showed that the discharge capability with the PPS-modified separator was higher than that of the routine separator.

The cycling performance of Li-S cells with the PPS-modified separator and with the routine separator were investigated at 0.2 C in Figure 6d. The cell with the PPS-modified separator delivered the discharge capacity of 855 mAh·g^−1^ and retained a high efficiency of more than 94% over 100 cycles. In addition, the cell with the routine separator only delivered 559 mAh·g^−1^ over 100 cycles. To further clarify the superior electrochemical performance of the cell with the PPS-modified separator, Figure 6e showed the rate performance from 0.1 to 3 C. It is worth noting, that the cell with the PPS-modified separator exhibited better capacity and delivered a high capacity of 507 mAh·g^−1^ even at 3 C. In contrast, the cell with the routine separator only delivered 163 mAh·g^−1^ at 3 C. The charge-discharge curves of the cell with the PPS-modified separator are presented at Figure 6f, showing a low potential gap between the charge-discharge plateaus voltage, and exhibit two flat typical discharge plateaus even at 3 C. The stability of the Li-S cells was further investigated by the long-term cycle, and the cell with the routine separator delivered a low capacity of 201 mAh·g^−1^ after 500 cycles. On the contrary, the cell with the PPS-modified separator exhibited excellent long-term cycling performance as high as 517 mAh·g^−1^ after 500 cycles at 1 C, suggesting superior long-term cycling stability. 

**Figure 7:**
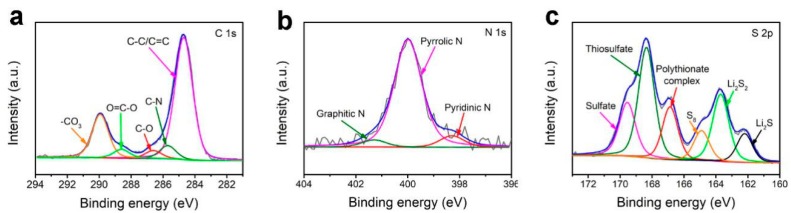
XPS spectra of (**a**) C 1s, (**b**) N 1s and (**c**) S 2p of the PPS coating layer after cycling.

In order to further demonstrate the adsorption of polysulfide by PPS, the XPS spectra of the cycled PPS coating layer was shown in Figure 7. As shown in Figure 7a, the C 1s spectrum was resolved into five peaks, corresponding to different chemical bonds of carbon in PPS. The spectrum of N 1s was shown in Figure 7b, the peaks at 401.2, 399.8, and 398.2 eV correspond to graphitic-N, pyrrolic-N, and pyridinic-N. As shown in Figure 7c, the S 2p XPS spectrum reveals six sulfur peaks. The three major peaks at 169.6, 168.4, and 166.9 eV were ascribed to sulfate, thiosulfate complex groups, and polythionate complex, respectively. In addition, two peaks at 163.7 and 162.2 eV could be assigned to the formation of Li_2_S_2_ and Li_2_S, respectively [43,44,45]. These results proved that the PPS coating layer could improve the capability of polysulfide absorption.

To compare the electrochemical performance of these interlayers or modified separators, further comparison among these Li-S batteries were carried out as shown in Table 1. It is significant to note that the cycling performance of the Li-S batteries with the PPS-modified separator exhibited a good cycle performance and rate capability.

## 4. Conclusions

In summary, a novel hierarchically porous polypyrrole sphere (PPS) was successfully obtained using as coating layer to restrain the shuttle effect of polysulfide. The mesopores in the PPS matrix reduce the mass density of the coating and act as a collector to adsorb polysulfides and enhance the utilization of sulfur. The Li-S cell with the PPS-modified separator delivered a high discharge capacity of 1274 mAh·g^−1^ and maintained the discharge capacity of 855 mAh·g^−1^ with a corresponding coulombic efficiency of 94% after 100 cycles. Moreover, the PPS-modified separator improved the polarization of the cell, exhibiting excellent rate performance. The developed strategy of using the PPS-modified separator was expected to improve the electrochemical performance of Li-S batteries.

## Figures and Tables

**Table 1:** Comparison of the electrochemical performance of previous reports with our work.

Sample	Sulfur Loading of Cathode (Content or Areal Loading)	Initial Capacity (mAh·g^−1^, at n C)	Final Capacity (mAh·g^−1^, after n Cycles)	High Rate Performance (mAh·g^−1^, at n C)	Ref.
Super P coated separator	1.5–2.0 mg·cm^−2^	~1000 mAh·g^−1^ (0.1 C)	610 mAh·g^−1^ (200 th)	~390 mAh·g^−1^ (1 C)	[46]
PPy modified separator	70%, 1.2 mg·cm^−2^	985 mAh·g^−1^ (0.5 C)	805 mAh·g^−1^ (250 th)	682 mAh·g^−1^ (2 C)	[47]
RGO/AC interlayer	58.2%	1078 mAh·g^−1^ (0.1 C)	655 mAh·g^−1^ (100 th)	348 mAh·g^−1^ (1.5 C)	[48]
N-PCNW- modified separator	1.5–1.7 mg·cm^−2^	1430 mAh·g^−1^ (0.2 C)	881.5 mAh·g^−1^ (200 th)	618 mAh·g^−1^ (2 C)	[49]
PrNPs	70%, 1.1 mg·cm^−2^	986 mAh·g^−1^ (0.2 C)	695 mAh·g^−1^ (200 th)	753 mAh·g^−1^ (2 C)	[50]
ZnO/CNT/RGO interlayer	68.3%, 1.7 mg·cm^−2^	1061 mAh·g^−1^ (0.2 C)	768 mAh·g^−1^ (200 th)	597 mAh·g^−1^ (2 C)	[51]
PPS-modified separator	2.1 mg·cm^−2^	1274 mAh·g^−1^ (0.2 C)	855 mAh·g^−1^ (100th)	507 mAh·g^−1^ (3 C)	This work
